# Digital imaging and qPCR analysis and comparison of short-term plaque removal effects of toothbrushing

**DOI:** 10.3389/fdmed.2023.1103602

**Published:** 2023-03-14

**Authors:** Yuanyuan Luo, Danni Wang, Yuxiao Li, Dezhi Geng, Bei Yu, Yueping Zhao, Qi Xiang

**Affiliations:** ^1^ Department of Stomatology, Liaocheng People's Hospital, Liaocheng, China; ^2^ Biopharmaceutical Research Development Center of Jinan University, Jinan University, Guangzhou, China; ^3^ Jinan University School of Stomatology, Jinan University, Guangzhou, China; ^4^ Institute of Biomedicine and Guangdong Provincial Key Laboratory of Bioengineering Medicine, Jinan University, Guangzhou, China

**Keywords:** dental plaque, digital image analysis, cariogenic bacteria, toothbrush, oral irrigator, real-time fluorescent quantitative polymerase chain reaction

## Abstract

**Purpose:**

Digital imaging technology and a real-time fluorescent quantitative polymerase chain reaction (RQ-PCR) were used to determine the changes in dental plaque caused by different toothbrushing tools.

**Methods:**

A total of 120 subjects were selected and divided into four groups: a manual toothbrush group, a manual toothbrush combined with an oral irrigator group, an electric toothbrush combined with an oral irrigator group, and an electric toothbrush group. We compared the changes in plaque count, plaque area, and colony colonization of the four groups after different cleaning tools had been used for a period of time.

**Results:**

Dental plaque count and plaque area decreased in all four groups. The decreases in plaque count and *Streptococcus mutans* in the electric toothbrush combined with an oral irrigator group were significantly higher than those in other groups.

**Conclusion:**

Electric toothbrush combined with an oral irrigator shows a good result for plaque removal effect. Digital image analysis combined with biological methods can be used to evaluate dental plaque.

## Introduction

Dental caries and periodontitis, as the two most prevalent oral diseases, are closely related to dental plaque. Plaque microorganisms on the tooth surface can produce acid and lead to the formation of dental caries and also act as the initiating factor of periodontitis. It can also cause the absorption and attachment loss of alveolar bone, and eventually lead to tooth loosening and falling off. Due to the lack of tissue repair, the destruction of the tooth and periodontal tissue becomes irreversible. Therefore, “prevention is more important than treatment” should be the principle adhered to by dentists (1). It can be seen that it is more urgent to remove dental plaque to prevent the occurrence of caries and periodontitis than related treatment, and brushing is the most important and effective measure to remove dental plaque and also the most important means for individuals to self-remove plaque and prevent dental caries and periodontitis.

At present, manual toothbrushes and powered toothbrushes are widely used; however, toothbrushes can only clean the food residues and plaques attached to the surface of teeth, and it is difficult to clean food residue and plaque located in deep crevices (2). To mitigate the disadvantages of toothbrushes, oral irrigators were invented. These can reach the hidden places of the oral cavity through high-pressure pulsed ultra-fine water columns generated by a pulsed pump body. As an auxiliary brushing tool, the irrigator became gradually popular and well-used. Christoph et al. (3) showed that after 8 weeks of brushing, the plaque removal effect on gingivitis patients who used an electric toothbrush combined with an oral irrigator was better than that of those who used a manual toothbrush. Costa et al. (4) showed that using a manual toothbrush combined with an irrigator can cause a bigger improvement in the periodontal state than using a manual toothbrush alone. Elkerbout et al. (5) suggested that electric toothbrushes are better at removing plaque than manual toothbrushes.

However, studies have also shown that there is no difference in plaque removal between electric and manual toothbrushes (6). As can be seen, there is still controversy regarding the benefits of each type of brushing tool.

In this study, digital image analysis technology combined with a real-time fluorescent quantitative polymerase chain reaction (RQ-PCR) was used to measure the changes in plaque area and flora colonization caused by different cleaning tools. The results provide a basis for quantifying the plaque-cleaning effect and guiding correct oral hygiene, as well as provide a reference for establishing a plaque evaluation system suitable for clinicians’ daily use.

## Materials and methods

### Study population

The study protocol was submitted to and approved by the Ethics Committee of the Biomedical Center of Jinan University (JNUKY-2021-013). A total of 120 volunteers were enrolled (the sample size was calculated based on randomized controlled clinical trials). A block random method randomly assigned the participants to the manual toothbrush group (group A), manual toothbrush combined with an oral irrigator group (group B), electric toothbrush combined with an oral irrigator group (group C), and electric toothbrush group (group D).

### Inclusion and exclusion criteria

The inclusion criteria were as follows: participants aged between 18–40 years; with good health and no systemic diseases; those that had at least 20 natural teeth; and with a plaque index of ≥1.5. Following screening, subjects were excluded if there were as follows: (1) women who were currently pregnant, lactating, or planning to become pregnant in the next 2 months; (2) patients with periodontal diseases; (3) patients who wore dental appliances; (4) those who at the time had five dental caries or mucosal lesions requiring treatment; (5) those with a history of allergy to any of the tools used in the study; (6) those taking drugs that could have an impact on the test results; and (7) those who had participated in oral clinical tests within 1 month.

### General design

We adopted the method of centralized training and on-site demonstration, making sure everyone had mastered the Bass brushing method. Participants brushed their teeth once a day in the morning for 3 min using the Bass brushing technique. The toothbrushing tools used were a Colgate manual toothbrush (soft, smart brush head), a Soshi electric toothbrush (sonic, fluffy, multimode), a Soshi irrigator (whole body waterproof, multi-gear mode, constant voltage pulse, and with pulse water flow usage), and a Chinese double calcium anticavity toothpaste. The participants returned every 7 days without brushing their teeth or gargling, and then the same doctor completed a lip/buccal plaque count (the modified Quigley-Hein plaque index was used to evaluate the plaque count). Plaque collection and plaque staining at specified time points (Day 0, Day 7, and Day 14) and the plaque area were analyzed by digital imaging technology. Since only the lip/buccal side plaque images of anterior teeth and premolars were easy to collect, the sum of effective anterior teeth and premolars lip/buccal side plaque index was assessed in this study. While only the detection of anterior teeth and premolar plaque data cannot completely record the changes in oral plaque, a real-time quantitative polymerase chain reaction (FQ-PCR) was used to detect the colonization of molar flora.

### Analysis of the change in plaque area with digital imaging technology

**Figure 1:**
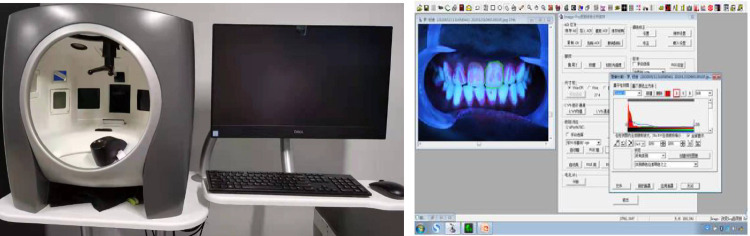
VISIA-CR image system.

**Figure 2:**
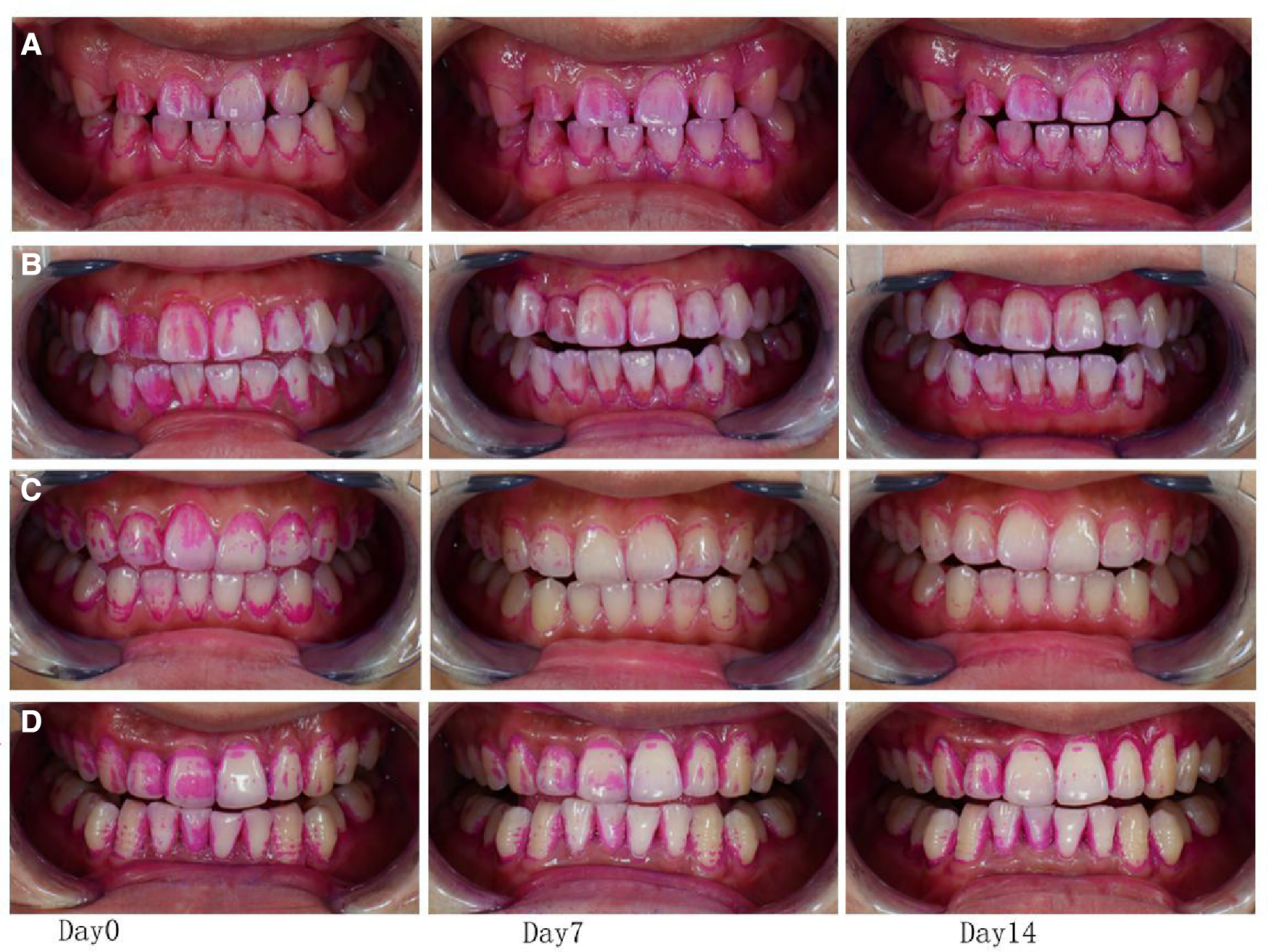
Image of plaque staining.

The VISIA-CR imaging system acquired plaque staining images, which were processed by the imaging software IPP 7.0 and quantitatively analyzed for changes in plaque area (Figures 1, 2). Specific operations procedures are as follows: open the image processing software, select “Skin Analysis” at the position where the collected images are opened in the file window, then select “Import AOI”, and select “Measure” after checking the target area. The results of plaque area comparison were expressed as a difference (*δ* = DN–D0) and the rate of plaque area decline expressed as follows: (Total plaque area of D0–D7/D14 Total plaque area/Total plaque area of D0) × 100%.

### Changes in molar microflora colonization detected by the real-time fluorescent quantitative polymerase chain reaction

In this study, a total of 48 subjects with 12 subjects in each group were selected. By scraping the buccal supragingival plaque of the subjects’ molars (first and second molars), extracting the plaque DNA, and detecting the colonization changes of *Streptococcus mutans*, *Streptococcus oralis*, *Actinomyces neisseria*, *Actinomyces caries*, and *Lactobacillus* in the plaque by RQ-PCR technology, we could better evaluate the plaque removal effect.

Before plaque staining, cotton wool was used to separate the dampness, and then a curler was used to scrape the dental plaque on the buccal gingiva of the first and second molars. The dental plaque was put into an EP tube filled with PBS and placed at −2°C for storage within 2 h. The samples were taken out and centrifuged, and the concentration and purity of DNA were extracted. The 1.5mlEP tube filled with DNA was sealed with a sealing membrane and placed at −8°C for storage. The specific primers were detected by conventional PCR, and primers (7, 8) were designed with reference to the relevant literature. The final primer selection was as shown for flora amplification and analysis of the amplification products. With a 2000 DNA marker as the measurement standard of the size of target gene fragments, all the amplification products showed specific bands consistent with the expected lengths of DNA fragments during primer design and synthesis. No specific amplification was conducted except for the target bands, suggesting that the primers had good specificity and could be used for the real-time fluorescent quantitative PCR reaction. For the fluorescence quantitative standard curve and dissolution curve formed by the amplification of genomic DNA of dental plaque bacteria, the dissolution curves presented sharp single peaks, indicating that the primers had good specificity, the amplification product was single, and there was no dimer. The linear correlations were all above 0.99, and the amplification efficiency was between 80% and 90%, indicating that the amplification efficiency was high. The target band was observed under ultraviolet light, and the main cariogenic bacteria were detected by PCR (refer to the standard operating procedure). The data were detected by Bio-Rad CFX manager 3.0, and the experimental results were analyzed by the normalized expression (2^−ΔΔCT^ Ct) method.

### Statistical analysis

All data in this study were statistically analyzed with GraphPad Prism 8.0 software, expressed as mean standard error (SEM), and the differences among the data of each group were compared with multi-factor analysis of variance (ANOVA). *P* < 0.05 was expressed as * indicating that the differences within the group were statistically significant, and *P *< 0.01 was expressed as ** indicating that the differences within the group were statistically significant. *P *< 0.05 was denoted by ^#^, and the difference between groups was statistically significant. *P *< 0.01 was denoted by ^##^ which meant that the difference between groups was statistically significant.

## Results

### Plaque count and plaque area analysis results

**Figure 3:**
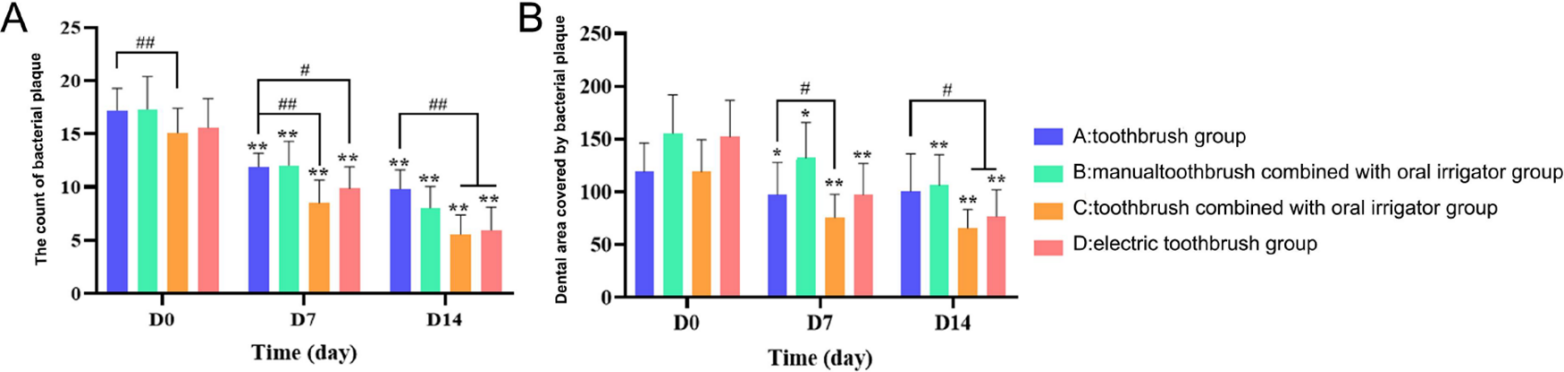
Labial/buccal plaque count (**A**) and plaque area bar chart of anterior teeth and premolars (**B**). The test data of the sample accord with the normal distribution. Intra-group differences were indicated by *P < 0.05 and **P < 0.01; The differences between groups were indicated by ^#^*P *< 0.05 and ^##^*P *< 0.01.

Compared with Day 0, the plaque count in the four groups decreased at time points Day 7 and Day 14, with statistical significance (*P *< 0.05). While group C decreased more significantly than other groups (*P *< 0.01), group D decreased more significantly than group A (*P *< 0.05), and group B did not show an advantage over group A although it did decline (Figure 3). Digital image analysis of plaque area showed that compared with Day 0, the plaque area of the four groups decreased at D7 and D14, with statistical significance (*P *< 0.05), while group C and group D decreased significantly (*P *< 0.01), with statistically significant differences. The decreased plaque area of group B showed an advantage over group A (Figure 3).

### Analysis of bacterial expression in molar plaque

**Figure 4:**
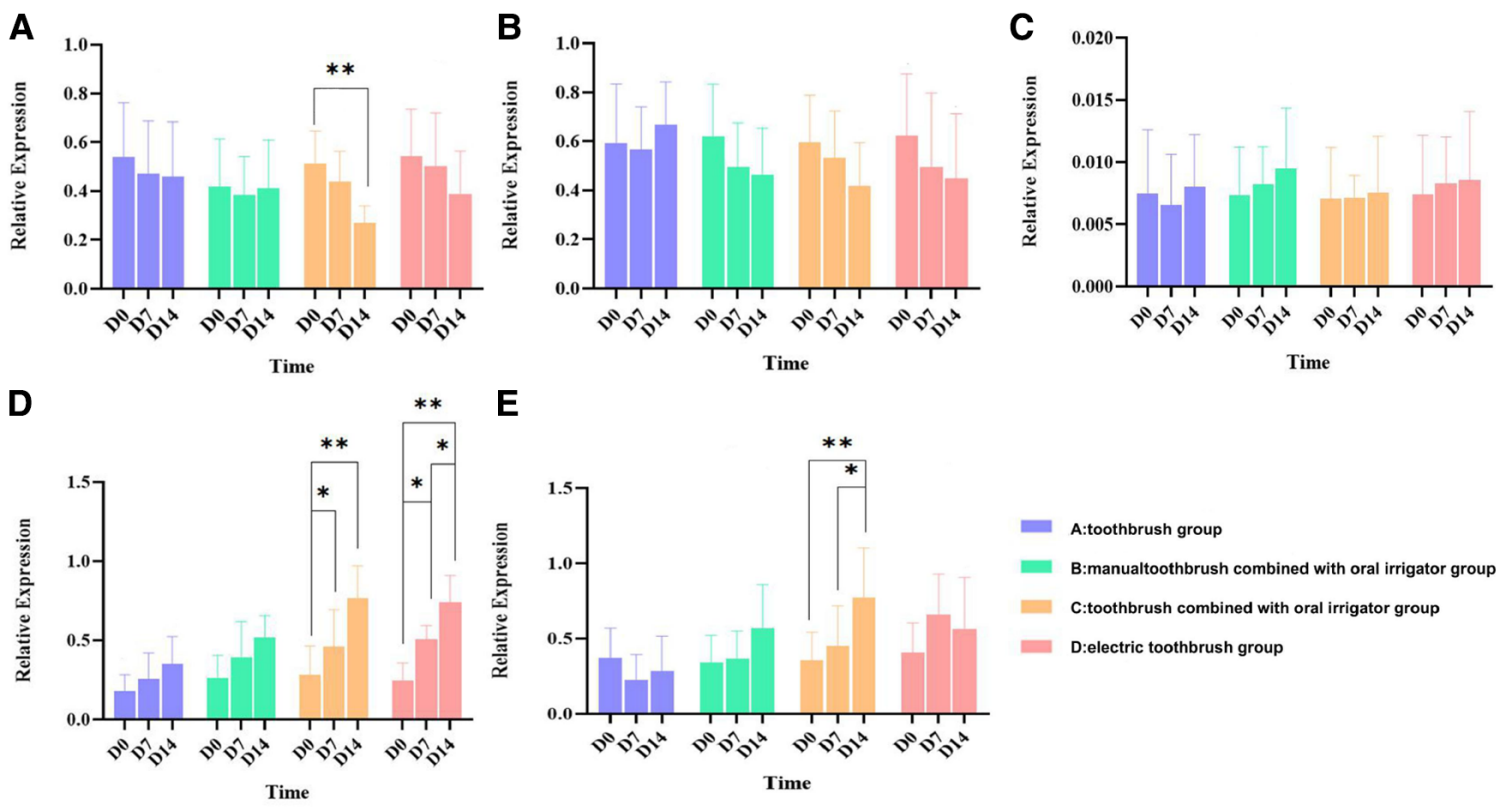
Bar chart of relative expression of bacteria in dental plaque. The test data of the sample conform to the normal distribution. *P < 0.05, the difference was statistically significant; **P < 0.01, the difference was statistically significant. (**A**) Streptococcus mutans; (**B**) oral streptococcus; (**C**) Lactobacillus; (**D**) Actinomyces odontolyticus; and (**E**) Actinomyces naeslundii.

During this study, *Streptococcus mutans* in groups C and D showed a continuous downward trend, and group C showed a significant difference (*P *< 0.01), while there was no significant change in groups A and B (*P *> 0.05). The expression level of Actinomycetes nesi showed a slight downward trend in group A, and other groups showed an upward trend, with the upward trend in group C being significant (*P* < 0.05). Oral streptococcus showed an upward trend in group A and other groups showed a downward trend, with the downward trend in group C being more obvious, but there was no statistical difference (*P *> 0.05) observed. The expression levels of *Actinomyces odontolyticus* in the four groups all presented an increasing trend, while the increasing trends in groups C and D were significant, with a statistical difference (*P* < 0.05). The expression levels of *Lactobacillus* in the four groups were low, ranging from (0.005–0.01) and showed no significant changes (*P* > 0.05) (Figure 4).

## Discussion

Digital imaging technology is an objective and sensitive method for evaluating dental plaque as it analyzes stained images to accurately quantify the plaque area without relying on the subjective feelings of the operator. At present, this method has been successfully applied to patients undergoing orthodontic treatment and can be repeatable (9, 10). Although digital imaging technology is simple and feasible in the evaluation of dental plaque in the front of the mouth, image acquisition in the molar area is costly and complicated. Due to the limited curvature of the arch, even when multiple images are taken from different angles, the tooth surface records are insufficient to effectively make an evaluation. Studies have shown that the plaque accumulation in the posterior teeth is greater than that in the anterior teeth. Evaluating plaque in the anterior region alone may underestimate the amount of plaque accumulation (11). Therefore, qPCR analysis of dental plaque on molars can better record the changes in dental plaque, so as to evaluate the plaque removal effect of different brushing tools.

*Streptococcus mutans*, oral *Streptococcus*, *Lactobacillus*, *Actinomyces odontolyticus*, and *Actinomyces naeslundii* in dental plaque are often detected and analyzed. *Streptococcus mutans* is considered the main cariogenic bacterium. Good cleaning practices can inhibit the growth of Streptococcus mutans and effectively prevent the occurrence of oral diseases (12). As one of the earliest colonized bacteria, oral *Streptococcus* has stronger adhesion than the later colonized bacteria, so it may be more difficult to remove during oral cleaning. The proportion of Lactobacillus in the whole oral flora is very small, usually about 0.01%–1% (13). Yang Ran et al. found that the detection rates of Actinomyces odontolyticus and Actinomyces naeslundii in a caries-free group were significantly higher than those in a caries-sensitive group, and speculated that *Actinomyces odontolyticus* and *Actinomyces naeslundii* may be caries-beneficial bacteria (14). Dame et al. showed that the expression of actinomycetes was very high in healthy teeth (15).

This research, based on different ways of oral cleaning, was conducted through digital imaging technology and a qPCR analysis of the plaque removal effect. The results for groups C and D showed that, after brushing teeth for a period of time, plaque area and plaque count in the expression of *S. mutans* had a declining trend, in either traditional plaque count, or plaque area of digital image analysis. The results were in agreement with the changes in plaque expression detected by qPCR. The decrease of plaque area and plaque count and *Streptococcus mutans* in the electric toothbrush combined with a dental flusher group was significantly higher than that in the other groups. These results may reflect that the brushing effect of an electric toothbrush combined with a dental flusher was better than that in the other groups.

A VISIA CR skin detector was used to register images of the participants’ mouths. It is one of up to 28 million skin analysis instruments. It uses built-in standard light, ultraviolet light, polarized light, and adjustable four kinds of xenon lamp light sources to accurately reproduce the color of the soft and hard tissue in the mouth, quickly generate a series of high-resolution images, and transform the image information into data for analysis. In addition, the VISIA CR's shooting system can record the position and angle of the first shot, and there will be a virtual head reminder for the second shot, ensuring that each shot is in the same position and angle, ensuring that the distance, light source, and angle of each shot are constant and uniform. The photographic tooth overlap degree is very high, up to 95%. In this way, the influence of operation (such as shooting distance, angle, camera shaking, etc.) on the shooting image and measurement results can be avoided, and the figure can clearly see the numerical change, which is convenient to guide the adjustment of treatment plans. This study found that compared with group A, the plaque area in group B decreased significantly, while the plaque count showed a downward trend, but there was no significant difference. Therefore, digital image analysis technology can accurately calculate complex and scattered plaque areas and has a stronger discrimination ability.

In conclusion, digital image analysis combined with biological methods can be used for the digital assessment of dental plaque in the whole mouth. In front teeth, digital image analysis is a reliable and effective method to measure dental plaque, and compared with the traditional plaque count evaluation method, digital imaging technology has the advantages of good repeatability and high sensitivity. In the rear teeth, due to the difficulty of image acquisition, biological detection is more convenient and accurate, and the risk assessment of caries, periodontitis, and other related diseases can be carried out in combination with the change of specific bacteria (this study did not carry out risk assessments due to time limitation). The combination of the two leads to the conclusion that the removal effect of plaque is better when an electric toothbrush is used in conjunction with an oral irrigator. Due to the short duration of this study (<3 months), and the requirements on brushing method, brushing time, and frequency, crossover experiments should be considered for a long-term clinical study in the future.

## Data Availability

The original contributions presented in the study are included in the article/Supplementary Material, further inquiries can be directed to the corresponding authors.
